# Impact of initial health assessment and crisis counselling for newly arrived Asylum seekers

**DOI:** 10.1192/j.eurpsy.2021.1942

**Published:** 2021-08-13

**Authors:** P. Uwamaliya

**Affiliations:** Faculty Of Health, Liverpool John Moores University, LIVERPOOL, United Kingdom

**Keywords:** Asylum seeker, mental health, Migration, Crisis Counselling

## Abstract

**Introduction:**

Evaluate the impact of the initial health assessment service for asylum seekers provided by the Asylum Practice Service.

**Objectives:**

Examine the inputs of Asylum practice service to asylum seekers. Investigate the activities and outputs of the Asylum practice service. Identify the outcomes of Asylum practice service to asylum seekers. Assess the impact of Asylum practice service to asylum seekers.

**Methods:**

The conceptual framework for measuring impact at the asylum practice service was based on a Logic Model to engage stakeholders and service users in order to evaluate the impact of services provided by the service. Also the Refugee Health Screener – 15 (RHS15) was used to screen the emotional distress/trauma to identify those individuals who would benefit from further mental health evaluation and treatment. Both quantitative and qualitative data were used to articulate and evidence social value performance and to tell the story of change created.

**Results:**

The study shows that newly arrived asylum seekers benefit from the services of asylum practice, even though the impact could be marginal in some cases.

**Conclusions:**

There is a need to revisit the current Initial Health Assessment tool, as in its current form, vital information on the causes of trauma such as rape, torture, human trafficking, and witnessing the death of parents, child, and close relatives which may underpin mental health problems, may not be captured, thus preventing access to appropriate interventions.

**Disclosure:**

No significant relationships.

